# An Account of the Practice of One of the Physicians of the Westminster General Dispensary, and of the Western Dispensary, from the 20th of July, to the 20th of August

**Published:** 1806-09

**Authors:** S. Fothergill

**Affiliations:** Southampton Street, Strand


*An Account of the Practice of one of the Physicians of the Westminster General Dispensary, and of the Western Dispensary, from the 20th of July, to the 20th of August.*

| ACUTE DISEASES. | |
|---|---|
| Measles - - - | 2 |
| Acute Rheumatism - | 6 |
| Peripneumony - - | 2 |
| Pleurisy - - - | 1 |
| Synochus - - - | 4 |
| Typhus - - - | 2 |
| Erysipelas - - | 1 |
| Catarrh - - - | 2 |
| Cholera - - - | 4 |
| Inflammation of the Parotid Gland - - | 1 |
| Acute Diseases of Infants | 9 |
| CHRONIC DISEASES. | |
| Asthenia - - | 9 |
| Cough and Dyspnœa | 12 |
| Pulmonary Consumption | 6 |
| Hæmoptoe - - | 2 |
| Chronic Rheumatism | 7 |
| ——— Ophthalmia - | 2 |
| Lumbago and Sciatica | 3 |
| Tic Douloureux - | 1 |
| Epilepsy - - - | 1 |
| Paralysis - - | 2 |
| Jaundice - - | 4 |
| Colic - - - | 2 |
| Diarrhœa - - - | 9 |
| Bilious Vomiting - | 2 |
| Gastrodynia - - | 8 |
| Dyspepsia - - | 4 |
| Scirrhous Liver - | 1 |
| Dropsy - - - | 2 |
| Cephalalgia and Vertigo | 6 |
| Hypochondriasis - | 3 |
| Worms - - - | 4 |
| Psora - - - | 2 |
| Prurigo Senilis - | 1 |
| Lichen Simplex - | 1 |
| Herpes - - - | 1 |
| Psoriasis Syphilitica | 1 |

Chlorosis

| | | | |
|---|---|---|---|
| Chlorosis - - | 1 | Leucorrhœa - - | 4 |
| Dysmenorrhœa - | 1 | Abortus - - - | 3 |
| Prolapsus Uteri - | 1 | Uterine Hæmorrhagy | 1 |

The summer has, hitherto, been favourable to health, though some individuals, more particularly exposed to the influence of the weather, have suffered from rheumatism and pulmonary affections, in consequence of the frequent rain and variable temperature. The thunder-storms and heavy showers in July, cleared the atmosphere of noxious particles, and caused a refreshing coolness, which in large towns, where a great portion of the population is excluded from fresh air, is highly beneficial.

Were shopkeepers, clerks, mechanics, and other descriptions of men, who are confined during the day, to walk abroad in the evening, instead of crowding together in taverns and ale-houses, summer complaints would diminish, and the moral and physical happiness of society materially improve. Games of every kind performed in the open air, and consistent with decency and propriety, should be encouraged; whilst they contribute to render the mind cheerful, they cherish the social affections, add vigour to the corporeal powers, and man thus rises in the scale of excellence.

Bathing, in particular, should be insisted upon; it strengthens the system, and by giving a gentle stimulus to the whole surface of the body, occasions a pleasurable excitement; the temperature is reduced to the natural standard, and cleanliness, a very certain promoter of health and comfort, is effected. Dispensary-Physicians, in their visits to the poor at their own dwellings, have ample opportunity to observe the consequences of long wearing the same clothes, loaded with dirt, and impregnated with animal matter; of living in close habitations, crowded with people, and swarming with vermin, where filth accumulates to a degree of insufferable offensiveness. These consequences cannot be too generally known; they lay the foundation for putrid diseases, the exciting cause has only to present itself and the victim is ready; the constitution enfeebled, offers no resistance; they multiply the miseries of the poor in health, and by their depressing debilitating power predispose to disease, which, under such circumstances, is always more fatal and alarming; children are born to perish before they are conscious of existence; and cutaneous diseases of every description swell the catalogue of evils. I have seen four children from one to seven years of of age, entirely naked, their bellies tumid, and their skin of a mud colour, lying upon a miserable mattrass on the floor, which at night also received the wretched parents.

In country towns, where the inhabitants enjoy the benefit of a running stream, men and boys are generally in the habit of frequent bathing; it is not unusual to see the numerous workmen of a large manufactory solacing themselves, after the labours of the day, in the limpid element. In a metropolis teeming with population, the boundaries being so immensely extensive, the difficulty of many people being able to go the necessary distance to the river, is obvious; and where every avenue is thronged with modest females, the gratification of a luxury, repugnant to delicacy, cannot be tolerated. This inculcates the necessity of public baths being established on liberal principles. There are already a sufficient number of baths for the use of the affluent; these, however, from their expence, are inaccessible to that class of people which is most in want of ablution, the call of hunger being much more imperious than the love of cleanliness. The want of so necessary an indulgence to the poor has been frequently urged, yet statesmen, who acknowledge that the strength of a nation consists less in the number than in the virtue and efficiency of the people, have hitherto neglected a very important means of rendering them more healthy, more cleanly, and of consequence more virtuous. We no longer live in an age when the magnificence of public works is derived from individual liberality; we do not now, as in ancient Rome, expect a man to rescue his name from oblivion by erecting stupendous aqueducts, or constructing baths, where, to use the language of Gibbon, "the meanest citizen could purchase with a small copper coin, the daily enjoyment of a scene of pomp and luxury, which might excite the envy of the kings of Asia." For the structure of such costly edifices we must, therefore, interest the philanthropy which so eminently distinguishes the opulent inhabitants of London; and when the utility of bathing is demonstrated, and the want of means to accomplish it is made known, doubtless, a subscription sufficiently ample may be raised, and the sanction of Government to an undertaking so patriotic, be obtained.

S. FOTHERGILL.

*Southampton Street, Strand, Aug. 21, 1806.*

*Account*

